# Co-administration of dexmedetomidine with total intravenous anaesthesia in carotid endarterectomy reduces requirements for propofol and improves haemodynamic stability: A single-centre, prospective, randomised controlled trial

**DOI:** 10.1097/EJA.0000000000002099

**Published:** 2024-11-11

**Authors:** Christian Vetter, Eva R. Meyer, Kathleen Seidel, David Bervini, Markus Huber, Vladimir Krejci

**Affiliations:** From the Department of Anaesthesiology and Pain Medicine, Inselspital, University Hospital, University of Bern, Bern, Switzerland (CV, ERM, MH, VK), and Department of Neurosurgery, Inselspital, University Hospital, University of Bern, Bern, Switzerland (KS, DB)

## Abstract

**BACKGROUND:**

Total intravenous anaesthesia guided by electroencephalography and neurophysiological monitoring may be used for carotid endarterectomy. Reduction of brain metabolic demand during cross-clamping of the internal carotid artery with propofol titrated to burst suppression requires effect-site concentrations that may delay emergence and interfere with intraoperative neurophysiological monitoring.

**OBJECTIVE:**

To test the hypothesis that dexmedetomidine decreases the effect-site concentration of propofol required for burst-suppression in patients undergoing carotid endarterectomy.

**DESIGN:**

Randomised controlled trial.

**PARTICIPANTS:**

Patients undergoing carotid endarterectomy.

**SETTING:**

University Hospital of Berne, Switzerland, from October 2018 to September 2024

**INTERVENTIONS:**

Patients were randomised into a control (*n* = 23) and a dexmedetomidine groups (*n* = 22). Total intravenous anaesthesia was administered to both groups. Patients in the dexmedetomidine group received an intravenous bolus of dexmedetomidine (0.4 μg kg^−1^ over 10 min) before induction, followed by a continuous intravenous infusion (0.4 μg kg^−1^ h^−1^). The effect-site concentrations of propofol were titrated against frontal electroencephalography parameters. Burst suppression was induced with propofol during cross-clamping of the internal carotid artery.

**OUTCOME MEASURES:**

The primary outcome was the effect-site concentration of propofol required for burst-suppression. The secondary outcomes were the requirement for vasoactive substances, neurophysiological monitoring parameters, and postoperative delirium.

**RESULTS:**

The effect-site concentration of propofol required for burst suppression was 4.0 μg ml^−1^ [3.50 to 4.90] (median [interquartile range]) in the dexmedetomidine group compared with 6.0 μg ml^−1^ [5.5 to 7.3] in the control group (*P* < 0.001). Less norepinephrine was required in the dexmedetomidine group (total 454 μg [246 to 818] compared with 1000 μg [444 to 1326] (*P* = 0.015) in the control group). Dexmedetomidine did not affect intraoperative neurophysiological monitoring.

**CONCLUSION:**

Co-administration of dexmedetomidine to total intravenous anaesthesia for carotid endarterectomy decreased the effect-site concentrations of propofol required for burst suppression by 33%. The propofol-sparing effect and peripheral alpha-agonism of dexmedetomidine may explain the reduced requirement for vasopressors.

**TRIAL REGISTRATION:**

Clinicaltrials.gov identifier: NCT04662177.

**KEY POINTS**

- An intravenous bolus (0.4 μg min^−1^ over ten minutes) before induction of anaesthesia for carotid endarterectomy, followed a continuous infusion (0.4 μg kg^−1^ min^−1^) decreased propofol requirements by 35%.
- In contrast to previous studies that used depth of anaesthesia (DoA) indices and proprietary algorithms, in this study propofol was titrated to raw EEG and spectral analysis criteria such as alpha-delta-power, or burst suppression.
- The propofol-sparing effect, as well as the alpha-agonistic properties of dexmedetomidine resulted in decreased requirements for norepinephrine.
- Adverse effects such as bradycardia were rare, and could be easily treated. The impact on intraoperative neurophysiology was clinically not relevant, and emergence was not delayed.

## Introduction

Stenosis of the internal carotid artery (ICA) is a risk factor for cerebrovascular incidents.^1^ Carotid endarterectomy (CEA) reduces the risk of future stroke in symptomatic and asymptomatic patients.^2,3^ General anaesthesia (GA), including total intravenous and locoregional anaesthesia, is equally well suited for CEA, with comparable outcomes.^4^ If intraoperative neurophysiological monitoring (IONM) is used to monitor cerebral perfusion, total intravenous anaesthesia (TIVA) is preferred since it interferes less with motor evoked potentials (MEP).^5,6^

Critical hypoperfusion may occur during ICA cross clamping. Oxygen delivery must be maintained under optimal cerebral perfusion pressure. Titration of propofol to EEG burst suppression (BS) is known to decrease the cerebral metabolic demand.^7,8^ In addition, several clinical and laboratory investigations suggest that propofol may be protective against ischaemia-reperfusion injury.^9,10^ However, the induction and maintenance of BS requires high concentrations of propofol, resulting in increased requirements for vasoactive drugs and delayed emergence.^11^

The centrally acting alpha-2 receptor agonist, dexmedetomidine, is mainly used for procedural sedation. Its use as an adjunct for GA during neurosurgical procedures, including CEA, has been reported to reduce anaesthetic requirements.^12–15^ Decreased risk of delirium and neuroprotective effects have also been suggested.^12,16–23^ Dexmedetomidine has a biphasic effect on arterial blood pressure (ABP); at higher concentrations, or when administered as an intravenous bolus, dexmedetomidine increases vascular resistance via peripheral alpha-adrenergic receptors. After prolonged administration and at lower concentrations, its central sympatholytic effects prevail.^24–26^ Indeed, during emergence and recovery from GA after CEA, co-administration of dexmedetomidine decreases the need for treatment of hypertension.^14^

There is some controversy regarding the effects of dexmedetomidine on the intraoperative neurophysiology. Although some authors have reported little or no effect, there are reports of significant interference.^7,13,27^

The main goal of this study was to test the hypothesis that co-administration of dexmedetomidine and propofol would result in a lower effect-site concentration (Cet) of propofol required for EEG BS. In addition, we investigated the effects of dexmedetomidine on haemodynamic, neurophysiology, and postoperative delirium.

## Methods

### Study design and participants

Ethical approval for this study (Ethical Committee No. NAC Project-ID: 2018-00220) was provided by the Ethical Committee NAC of Bern University Hospital, Bern, Switzerland (Chairperson, Prof. Dr med. C. Seiler) on 25 September 2018. This study has been registered at ClinicalTrials.gov (NCT04662177). Ninety-five participants were screened between October 2018 and September 2020. The inclusion criteria were ASA physical status ≤4, age ≥18 years, and symptomatic and asymptomatic stenosis of the ICA who undergo CEA. The exclusion criteria were: age <18 years, higher-grade atrioventricular block, severe hypovolaemia, bradycardia, uncontrolled hyper- or hypotension, hypersensitivity to dexmedetomidine, liver disease, known malignant hyperthermia, cardiovascular instability, or heart failure (NYHA ≥ 4), known limited peripheral autonomic activity, pregnancy, rejection, or lack of patient consent.

**Fig. 1:**
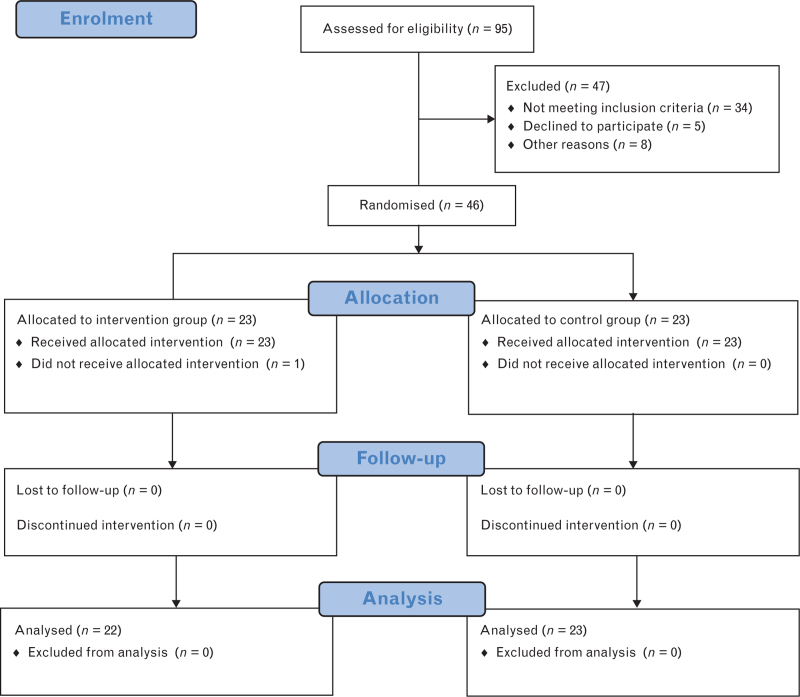
Patient flowchart.

Forty-six participants met the inclusion criteria (Fig. 1). After written consent was obtained, the participants were randomised into two groups of twenty-three participants: a control group and a dexmedetomidine group. Participants, surgeons and electrophysiology technicians were blinded for the allocation of the participant. The primary outcome was the effect site concentration (Cet) of propofol during BS. Secondary parameters were the haemodynamic effects of dexmedetomidine, defined as the total requirement for vasoactive substances, intravenous fluids, and urinary output. In addition, we recorded intraoperative neurophysiological parameters (amplitude of somatosensory evoked potentials (SEP) and stimulation thresholds of MEP), duration of anaesthesia and surgery, Glasgow Coma Scale (GCS), Richmond Agitation Sedation Scale (RASS), and criteria for the development of delirium using the confusion assessment method for the intensive care unit (CAM-ICU) on the first day following surgery.

### Anaesthesia

In the operating theatre, participants were monitored according to ASA standards (Philips Healthcare, Germany). Before induction, a radial artery catheter was inserted, and frontal EEG leads were applied and connected to an EEG monitor (Narcotrend, Germany).^27^ Before induction, the study group received dexmedetomidine as a bolus of 0.4 μg kg^−1^ over 10 min. Thereafter, dexmedetomidine was continuously infused at a rate of 0.4 μg kg^−1^ h^−1^ until the beginning of closure. Both groups received standard TIVA with propofol delivered by an automated target-controlled infusion (TCI) pump (Arcomed, Switzerland). The Cet of propofol was titrated using the model proposed by Schnider.^28^ Analgesia was achieved with intravenous boluses of fentanyl and continuous infusion of remifentanil (TCI, Minto). Rocuronium was administered intravenously to facilitate endotracheal intubation. No further neuromuscular-blocking agents were administered. Recovery was assessed (TOF Watch; Ireland) before the beginning IONM. After endotracheal intubation, the patients were connected to an anaesthesia ventilator (Primus, Dräger, Germany) and normoventilated.

**Fig. 2:**
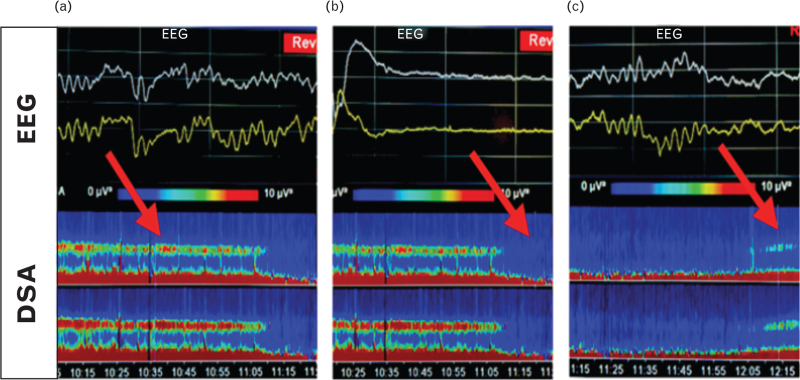
The raw EEG and the density spectral array during surgery. The raw EEG (top) and the density spectral array (DSA) (bottom) during anaesthesia before (a), during (b) and after burst suppression coma (c). (a) EEG under general anaesthesia before initiating burst suppression coma, showing alpha and delta waves. In the density spectral arrays (DSA) a clearly defined alpha and delta band, which is typical for propofol anaesthesia is visible. (b) EEG during burst suppression coma induced by increase in propofol. The DSA shows a preserved delta band and disappearance of the alpha band during burst suppression. (c) EEG after termination of the burst suppression at the end of surgery showing the recurrence of alpha and delta waves. The DSA shows the reappearance of the alpha band.

Propofol TCI was titrated individually to clinical and electroencephalographic endpoints (Fig. 2). Delta waves together with alpha oscillations in the frontal EEG were considered signs of adequate anaesthesia. Remifentanil TCI was adjusted to individual requirements, and additional fentanyl was administered as needed. Before cross-clamping the ICA, the Cet of propofol was gradually increased until a BS pattern appeared in the frontal EEG (Fig. 2b), with approximately one burst every ten seconds, which was also confirmed by the IONM technician.

### Haemodynamic management

Haemodynamic goals were defined individually based on the median of the last three documented blood pressure measurements before the patient was transferred to the operating theatre, and discussed with the surgical team before incision. Mean arterial blood pressure (MAP) was maintained as close as possible to the preoperative value. Before cross-clamping the ICA, MAP was increased by 20 mmHg. Before reperfusion, the systolic arterial blood pressure was decreased to 100–140 mmHg to avoid hyperperfusion syndrome.^29^

Normovolaemia was maintained using an intravenous lactated Ringer's solution. Norepinephrine was administered as a continuous infusion, and additional boluses of norepinephrine (5–10 μg), ephedrine (5–10 mg), or phenylephrine (50–100 μg) were administered intravenously, as needed. Bradycardia was treated using intravenous anticholinergic agents.

After reperfusion of the ICA, the Cet of propofol decreased to the level before BS and was further titrated according to the EEG (Fig. 2c). In the study group, dexmedetomidine was discontinued at the beginning of skin closure.

### Intraoperative neurophysiological monitoring

MEP and SEP were monitored (Inomed, Germany). For SEP, the median and posterior tibial nerves were stimulated with monopolar needles on bilateral using a square wave pulse of 200 μs duration, a stimulation of 1.7–4.7 Hz, and an intensity of 10–25 mA. Recording was performed on the scalp using corkscrew electrodes. For MEP, anodal constant-current stimulation was performed on the scalp using cork-screw electrodes with a train of 5 stimuli, 0.5 ms pulse duration and an interstimulus interval of 4.0 ms. Recording was performed with pairs of monopolar needles separated from each other by 1–2 cm, over at least the abductor pollicis brevis and tibialis anterior muscles.^27^

### Postoperative care and assessment of delirium

After surgery, TIVA was discontinued. Following extubation, the patients were transferred to an intermediate care unit, where they received standard care. Data acquisition was discontinued, except for the assessment of delirium on the first postoperative day using the CAM-ICU score.^30^

### Statistical analysis

The sample size was calculated as follows: assuming a Cet of 6 mg ml^−1^ with a standard deviation (SD) of 3 mg ml^−1^ in the control arm and a minimal clinically important difference of 3 mg ml^−1^ and an alpha of 0.05, at least twenty-three participants per group were required to reach a power of 90%. Continuous variables were assessed for normality using the Shapiro–Wilk test and were summarised as mean and standard deviation when normally distributed, and as median and interquartile range [IQR]. Categorical variables were summarised as counts and percentages. Group comparisons were based on the χ^2^-test (categorical variables), Student's *t*-test (normally distributed continuous variables), or Wilcoxon rank-sum test (including the primary outcome). Group differences in baseline data (characteristics and comorbidities) were based on standardised mean differences (SMD). No adjustment for multiple comparisons was deemed necessary, as there was only a single primary outcome and the secondary outcomes were considered exploratory. The missing data are indicated in the respective tables. Statistical significance was set at *P* < 0.05. All calculations were performed using R, version 4.0.2.^31^

## Results

Ninety-five participants were screened between October 2018 and September 2020. Forty-six patients met inclusion criteria. Twenty-three participants in the control group (CG) and twenty-two participants in the dexmedetomidine group (DG) were analysed. Owing to a medical decision, surgery was not performed in a patient in the DG after obtaining written informed consent and the patient was excluded. There were no other exclusions or participants who were lost to follow-up. After reaching the calculated sample size, the study was stopped. There were no differences between the two groups in baseline characteristics (Table 1) or in the duration of anaesthesia, surgery, and cross-clamping of the ICA or BS (Table 2).

**Table 1:** Patients’ characteristics

	Control group *n* = 23	Dexmedetomidine group *n* = 22	SMD
Age (years)	74.1 ± 9.6	75.5 ± 7.2	0.17
Sex (female)	*8* (34.8)	*5* (22.7)	0.27
Weight (kg)	77.4 ± 13.7	78.2 ± 13.3	0.06
Height (m)	1.69 ± 0.08	1.70 ± 0.09	0.03
Body mass index (kg m^−2^)	26.8 ± 3.7	27.1 ± 3.4	0.06
ASA physical status			0.36
3	*3* (13.0)	*6* (27.3)	
4	*20* (87.0)	*16* (72.7)	
Systole (mmHg)	146 ± 24.4	141 ± 21.8	0.23
Diastole (mmHg)	75.7 ± 11.3	69.8 ± 6.9	0.63
Hypertension	*18* (78.3)	*21* (95.5)	0.53
Coronary heart disease	*5* (21.7)	*8* (36.4)	0.33
History of myocardial infarction	*2* (8.7)	*1* (4.6)	0.17
Stroke <30 days	*15* (65.2)	*15* (68.2)	0.06
Asymptomatic stenosis of the ICA	*2* (8.7)	*6* (27.3)	0.5
COPD	*6* (26.1)	*3* (13.6)	0.32
Renal insufficiency	*9* (39.1)	*9* (40.9)	0.04
Diabetes mellitus	*3* (13.6)	*6* (27.3)	0.34
Alcoholism	*8* (34.8)	*5* (22.7)	0.27
Chronic smoking	*13* (56.5)	*9* (40.9)	0.32

Data are presented as *n* (%), mean ± SD, or median [IQR] as appropriate. CAD, coronary heart disease; COPD, chronic obstructive pulmonary disease; ICA, internal carotid artery; SMD, standardised mean difference.

**Table 2:** Intra-operative characteristics

	Control group *n* = 23	Dexmedetomidine group *n* = 22	*P*	*n*
Intra-operative measurements				
Duration of anaesthesia (min)	191 [180 to 208]	200 [177 to 213]	0.928	45
Duration of surgery (min)	108 ± 17.8	107 ± 25.0	0.820	45
Onset of BS until BS coma (min)	8.52 ± 4.4	7.75 ± 3.4	0.535	41
Duration of BS coma (min)	49.0 [45.0 to 51.5]	47.5 [40.5 to 57.8]	0.820	45
Clamping of internal carotid artery (min)	33.0 [31.0 to 37.0]	36.5 [32.2 to 40.0]	0.133	45
Duration of suture until extubation (min)	20.0 [13.5 to 23.5]	21.5 [11.2 to 31.8]	0.503	45
Primary outcome				
Propofol (Cet) (μg ml^−1^) during BS	6.00 [5.5 to 7.3]	4.00 [3.5 to 4.9]	<0.001	45
Secondary outcome				45
Norepinephrine (μg)	1000 [444 to 1326]	454 [246 to 818]	0.015	45
Propofol (mg)	1426 [1204 to 1782]	1158 [1002 to 1318]	0.013	45
Other drugs used during surgery				
Remifentanil (μg)	3012 ± 1001	2591 ± 964	0.158	45
Fentanyl (μg)	300 [200 to 400]	250[200 to 300]	0.406	45
Atropine (mg):			0.109	45
0	23 (100)	19 (86.4)		
0.5	0 (0.0)	2 (9.1)		
1	0 (0.0)	1 (4.6)		
Ephedrine (mg)	5.00 [0.00 to 10.0]	5.00 [0.00 to 10.0]	0.396	45
Epinephrine (μg):			1.000	45
0	21 (91.3)	22 (100)		
18	1 (4.4)	0 (0.0)		
82	1 (4.4)	0 (0.0)		
Infusion and urinary output during surgery				
Lactated Ringer's infusion (ml)	2000 [1225 to 2100]	1200 [1100 to 1875]	0.100	44
Urinary output (ml)	500 [150 to 900]	600 [350 to 950]	0.520	34

Data are presented as *n* (%), mean ± SD, or median [IQR] as appropriate. BS, burst suppression.

### Propofol requirements

The Cet of propofol required for BS were 4.0 μg ml^−1^ [3.50 to 4.90] (median [IQR]) in the DG compared with 6.0 μg ml^−1^ [5.5 to 7.3] in the CG, *P* < 0.001. The total amount of propofol administered was 1158 mg [1002 to 1318] in the DG and 1426 mg [1204 to 1782] in the CG (*P* = 0.013).

### Requirements for vasoactive substances

**Fig. 3:**
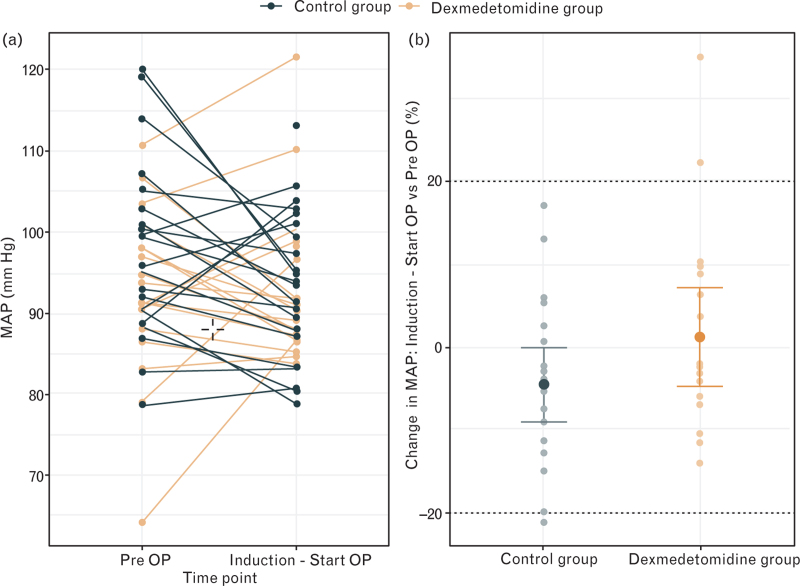
Pooled mean arterial blood pressure. Pooled mean arterial blood pressure (MAP) data of the study participants divided into the control group (black) and dexmedetomidine group (yellow). The blood pressure values before surgery were compared with the blood pressure values up to the start of surgery (Panel a). The ratio between the same MAP values from both groups shows that the MAPs of the control group tend to decrease between induction of anaesthesia and start surgery and those of the dexmedetomidine group roughly correspond to the preoperative values (Panel b).

**Fig. 4:**
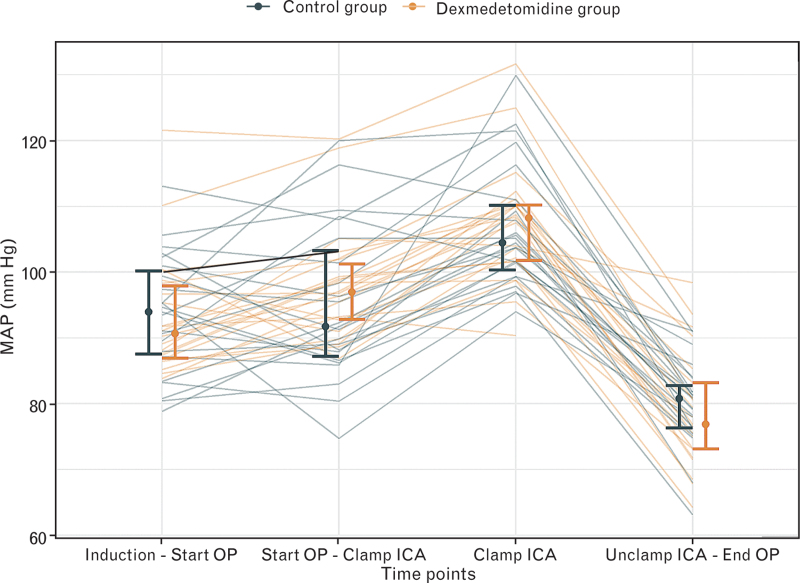
Blood pressure as mean arterial pressure (MAP) during different stages of the procedure for both groups. All MAP values measured at the four different timepoints were pooled and are illustrated in the graph: control group (black) and dexmedetomidine group (yellow). Individual patients are shown as solid lines; summary measures (median and interquartile range) are shown for each timepoint. The first stage is defined as the time from induction of anaesthesia until start of the procedure, the second from start of the procedure until cross-clamping of the internal carotid artery (ICA), the third from beginning to end of the cross-clamping of the ICA and the fourth from unclamping the ICA until the end of the operation. At none of the four stages was there a significant difference in the median MAP shown between the two groups. The elevation at the third stage reflects our haemodynamic protocol, which included an increase in MAP by 20 mmHg from the baseline blood pressure: mostly this was achieved by the continuous use of vasopressors. A decrease of systolic arterial blood pressure is shown at the fourth stage, to achieve a target systolic blood pressure between 140 and 100 mmHg.

Haemodynamic goals were achieved with less norepinephrine in the DG (total 454 μg [246 to 818] compared to the CG 1000 μg [444 to 1326] (*P* *=* 0.015; Table 2)). Pooled MAP values from different stages of anaesthesia and surgery are presented in Fig. 3a) and b). After induction of anaesthesia, MAP remained within ±20% of preoperative baseline values in all patients. During the different stages of surgery, there were no differences between the groups (Fig. 4).

The requirements for ephedrine, atropine, or epinephrine did not differ between the groups. Bradycardia was observed in three participants in the DG and one in the CG. One participant in the CG required repeated small boluses of epinephrine (total 100 μg) to maintain the ABP. No substantial blood loss was observed during surgery (data not shown). The amount of crystalloids and urine output were comparable between the groups (Table 2).

### Intraoperative neurophysiology

Overall, there were no clinically significant differences in the MEP and SEP between the groups. At baseline, the abductor pollicis brevis MEP stimulation thresholds (affected side; average, 95% CI) were 92 mA (78 to 106 mA) in the dexmedetomidine group and 107 mA (76 to 138 mA) in the control group. Median nerve SEP amplitudes on the affected side were, as average (CI): 3.0 (2.2 to 3.8) μV in the dexmedetomidine group, and 2.8 (1.9 to 3.7) μV in the control group. There were no differences between the two groups and also in comparison of the affected and the unaffected side. The SEP remained stable bilaterally in both groups. Although MEP thresholds slightly increased for both the affected and unaffected sides in both groups by 10–20% (notably after induction of burst suppression), their use for the detection of ischaemia remained intact. In one patient in the dexmedetomidine group, a temporary shunt had to be inserted after MEP and SEP were significantly altered immediately after cross-clamping. After insertion of the temporary shunt, the potentials recovered and remained stable together with good neurological outcomes.

### Postoperative assessment of delirium

The GCS score was 15 in forty-three of the patients on the first postoperative day. Postoperative cognition in two patients in the CG was transiently impaired, with a GCS score of 14. One of these patients had a history of benzodiazepine abuse and developed delirium, possibly because of withdrawal in the postoperative setting. The RASS score was zero in both the groups.

## Discussion

The primary outcome and main finding of this prospective randomised trial was that dexmedetomidine decreased the Cet of propofol required for BS by 33%. Second, hemodynamic goals were achieved with 50% less norepinephrine. Third, the co-administration of dexmedetomidine appeared to have few adverse effects on evoked potentials. Finally, we did not observe an increase in clinically relevant side effects, delayed emergence, or postoperative delirium.

The rationale for an initial bolus of 0.4 μg kg^−1^ over 10 min followed by a continuous infusion of 0.4 μg kg^−1^ h^−1^ was based on our clinical experience regarding haemodynamic stability, propofol requirements, emergence from anaesthesia, and side-effects, as well as on pharmacokinetic considerations. Several pharmacokinetic and pharmacodynamic models have been proposed since the introduction of dexmedetomidine (DEX). Depending on the choice of the model, predicted plasma concentrations (Cp) for our protocol ranged between 0.35 and 1 ng ml^−1^ following the initial bolus. During steady state conditions the models predicted a Cp between 0.3 and 0.5 ng ml^−1^.

Decreased requirements for propofol with co-administration of dexmedetomidine^16,18^ have been reported for loss of consciousness,^18^ blunting of noxious stimuli,^17^ or for bispectral index-guided titration of propofol for general anaesthesia^15,32,33^ in healthy volunteers, and ASA class 1 and two patients undergoing elective surgery.^15,17,18,32,33^ Initial boluses of 0.3–1.0 μg kg^−1^, followed by a continuous infusion ranging from 0.3–0.6 μg kg^−1^ h^−1^, or TCI-algorithms targeting plasma concentrations between 0.2 and 0.8 ng ml^−1^were applied.^18,32^ Reported propofol-sparing-effects were between 30% and 50% and were dose-dependent.^32^ Le Guen found that an initial bolus of 1 μg kg^−1^ of dexmedetomidine followed by 0.5 μg kg^−1^h^−1^ reduced propofol requirements by 30% for induction and maintenance of anaesthesia using an automated closed loop algorithm.^15^ Similar regimens resulted in a 35–37% reduction of propofol requirements during endoscopic retrograde cholangiopancreatography and breast surgery.^33^ Ge *et al.*^1,12^ reported a 10% decrease of propofol requirements during CEA under GA when dexmedetomidine was co-administered as an initial bolus of 0.3 μg kg^−1^ followed by 0.3 μg kg^−1^ h^−1^.

In all of the studies mentioned above, titration of propofol was guided either by the bispectral index or by clinical presentation. To the best of our knowledge, the present study is the first to quantify the propofol-sparing effects of dexmedetomidine using electroencephalographic waveforms and spectral analysis, as described by Purdon *et al.*^34^ In brief, propofol-induced unconsciousness was associated with coherent alpha oscillations in thalamic-cortical circuits. Dexmedetomidine results in an electroencephalographic pattern similar to that of nonrapid eye movement sleep, that is, slow (delta) oscillations and spindles. These appear in frontal leads and can be displayed as raw EEG and spectrograms with drug-specific ‘signatures’. BS is a reproducible EEG pattern that can be easily recognised.

Our study included patients with ASA classes 3 and 4, the majority of whom had symptomatic ICA stenosis or a recent ischemic stroke. Cerebral autoregulation may be impaired in high-risk populations. Therefore, the cerebral perfusion pressure must be tightly controlled to avoid ischaemia or hyperperfusion syndrome. To date, few studies have investigated the use of dexmedetomidine during GA for carotid endarterectomy,^1,12,14^ but most have reported improved haemodynamic stability. Our findings showed that patients in the DG required significantly less norepinephrine to achieve haemodynamic targets, which was partly due to decreased propofol requirements. In addition, dexmedetomidine produces dose-dependent, nonlinear vasoconstriction mediated by peripheral alpha-2_b_ receptors.^24,25,35^ Recently published trials have reported that the use of dexmedetomidine in patients with sepsis might decrease the requirement for vasoactive support.^36^ To our knowledge, no study has been published on its intraoperative use.

The action of dexmedetomidine on central alpha-2_a_ receptors blunts the neuroendocrine stress response in various settings including cardiac, abdominal, and neurological surgeries. In a study by Tsujikawa *et al.*,^14^ dexmedetomidine was administered only after carotid reperfusion, resulting in improved haemodynamic stability during emergence and recovery and a decreased need for antihypertensive therapy, which reduced the risk of hyperperfusion syndrome.^14,37^ Similar to our study, the incidence of bradycardia requiring treatment was not increased by dexmedetomidine. Although the propofol-sparing effect of dexmedetomidine did not result in faster recovery, emergence from anaesthesia was not delayed. Before transfer to the ICU, both groups showed favourable outcomes for GCS and RASS and comparable preoperative and postoperative National Institutes of Health Stroke Scale scores.

Several studies have reported a reduction in the incidence of postoperative delirium when dexmedetomidine is used, as well as neuroprotective effects.^19,20,38^ Ge *et al.* reported enhanced recovery and cognition associated with decreased levels of markers of cerebral damage, antioxidative effects ^1^ and inflammatory cytokines.^12^ Decreased pro-inflammatory cytokines, apoptotic signalling pathways, and the formation of oxygen-free radicals after ischaemia or reperfusion injury have been reported in animal studies, indicating that neuroinflammation could play a role in cognitive dysfunction. However, the exact mechanism and its clinical importance remain unclear.^12,21,22,39^ A reduction in the incidence of postoperative delirium was found in patients undergoing noncardiac surgery who received a low dose of dexmedetomidine.^20^ However, that study protocol excluded participants undergoing neurosurgical procedures, and infusion of dexmedetomidine was initiated only after arrival in the ICU. In the present study, we found no significant differences in neurological outcomes, possibly because the study was neither designed nor powered. Delirium was assessed on the first postoperative day only, and there were no further neuropsychological tests, such as the Mini-Mental State Examination.

Several trials have suggested an association between deep anaesthesia and postoperative delirium; however, causality has not yet been established. In the present study, two patients in the CG had delayed neurological recovery. However, in both cases, representing less than 5% of the study population, aetiologies other than BS may have been involved. One patient in the CG with likely postoperative delirium had a history of benzodiazepine abuse, and in the other patient, hemineglect was present before surgery. Although the present study is likely to be underpowered for detection or exclusion of delirium, the previously reported incidence of delirium of up to 30% is six-fold higher than that observed in this frail, high-risk population. A previously published study on patients undergoing CEA at the Department of Neurosurgery of the University Hospital of Bern routinely reported excellent outcome^8^ with our anaesthetic protocol.

Volatile agents and propofol at higher concentrations are known to affect intraoperative neurophysiological evoked potentials in a dose-dependent manner.^40–43^ The effects of dexmedetomidine on evoked potentials have been investigated, but the results are controversial. In spine surgery, dexmedetomidine has shown little effect on MEP and SEP^13,44,45^ and our study found no clinically significant adverse effects of dexmedetomidine on MEP and SEP. However, the study was not sufficiently powered to answer this question.

### Strengths and limitations of the study

This prospective, randomised controlled study was conducted in a clinical setting, with surgical and anaesthesia protocols applied as in everyday routine. Thus, our findings are likely to be applicable to clinical practice, which is one of the main strengths of this study.

One of the limitations is the lack of blinding of the anaesthesiologist to dexmedetomidine. Although this may have introduced some bias, it is unlikely that our main findings were significantly affected. First, BS is a reproducible electroencephalographic pattern that was confirmed by a neurophysiology technician who was not aware of the allocation. Furthermore, haemodynamic goals were defined by preoperative parameters and always discussed with the surgical team, who was blinded. Thus, the dosage of propofol for electroencephalography and norepinephrine for haemodynamic goals is likely driven by objective and reproducible parameters.

Currently, the relationship between BS and delirium is subject to controversy. The diagnosis of delirium is complex and requires repeated neurological assessments over several days after surgery. Although delirium was defined as a secondary endpoint, the present study would have been underpowered to provide a comprehensive answer. Moreover, collection of data was discontinued on the first day following surgery.

In addition, there were no differences in intravenous fluids, and as CEA usually results in minimal blood loss, it is unlikely that individual choices in fluid management significantly affected the results given the median effect size of 33% for propofol and 50% for norepinephrine.

The cerebral hyperperfusion syndrome (CHS) is a rare but serious complication after cerebral revascularisation procedures. It is associated with high arterial blood pressure after reperfusion and characterised by headaches, neurological deficits, and seizures not caused by cerebral ischaemia. The present study was designed and powered to detect differences in concentrations of propofol, and does not allow to make statements concerning CHS. This is another limitation of our study.

## Conclusion

Co-administration of dexmedetomidine with TIVA for CEA resulted in a significant reduction in the Cet required for EEG-guided general anaesthesia and BS. The propofol-sparing and peripheral alpha-agonistic properties of dexmedetomidine likely explain why less norepinephrine was required to maintain ABP within the defined range. Furthermore, with reduced amounts of propofol, dexmedetomidine did not appear to have clinically significant adverse effects on neurophysiological monitoring.

## References

[1] Global, regional, and national burden of stroke and its risk factors, 1990–2019: a systematic analysis for the Global Burden of Disease Study 2019. Lancet Neurol 2021;**20**:795–820. 10.1016/S1474-4422(21)00252-0 PMC8443449 34487721

[2] Chambers BR Donnan GA. Carotid endarterectomy for asymptomatic carotid stenosis. *Cochrane Database Syst Rev* 2005; 2005:Cd001923. 16235289 10.1002/14651858.CD001923.pub2 PMC6669257

[3] Rerkasem A Orrapin S Howard DP Rerkasem K. Carotid endarterectomy for symptomatic carotid stenosis. *Cochrane Database Syst Rev* 2020; 9:Cd001081. 32918282 10.1002/14651858.CD001081.pub4 PMC8536099

[4] Gough MJ Bodenham A Horrocks M. GALA: an international multicentre randomised trial comparing general anaesthesia versus local anaesthesia for carotid surgery. *Trials* 2008; 9:28. 18495004 10.1186/1745-6215-9-28 PMC2413207

[5] Malcharek MJ Ulkatan S Marinò V. Intraoperative monitoring of carotid endarterectomy by transcranial motor evoked potential: a multicenter study of 600 patients. *Clin Neurophysiol* 2013; 124:1025–1030. 23200315 10.1016/j.clinph.2012.10.014

[6] Macdonald DB Skinner S Shils J Yingling C. Intraoperative motor evoked potential monitoring – a position statement by the American Society of Neurophysiological Monitoring. *Clin Neurophysiol* 2013; 124:2291–2316. 24055297 10.1016/j.clinph.2013.07.025

[7] Müller MD Seidel K Peschi G. Arterial collateral anatomy predicts the risk for intra-operative changes in somatosensory evoked potentials in patients undergoing carotid endarterectomy: a prospective cohort study. *Acta Neurochir (Wien)* 2021; 163:1799–1805. 33099692 10.1007/s00701-020-04624-y PMC8116285

[8] Reinert M Mono ML Kuhlen D. Restenosis after microsurgical nonpatch carotid endarterectomy in 586 patients. *Acta Neurochir (Wien)* 2012; 154:423–431. discussion 431. 22113556 10.1007/s00701-011-1233-9 PMC3284671

[9] Madathil RJ Hira RS Stoeckl M. Ischemia reperfusion injury as a modifiable therapeutic target for cardioprotection or neuroprotection in patients undergoing cardiopulmonary resuscitation. *Resuscitation* 2016; 105:85–91. 27131843 10.1016/j.resuscitation.2016.04.009

[10] Hausburg MA Banton KL Roman PE. Effects of propofol on ischemia-reperfusion and traumatic brain injury. *J Crit Care* 2020; 56:281–287. 32001426 10.1016/j.jcrc.2019.12.021

[11] Pawar N Barreto Chang OL. Burst suppression during general anesthesia and postoperative outcomes: mini review. *Front Syst Neurosci* 2021; 15:767489. 35069132 10.3389/fnsys.2021.767489 PMC8776628

[12] Ge Y Li Q Nie Y. Dexmedetomidine improves cognition after carotid endarterectomy by inhibiting cerebral inflammation and enhancing brain-derived neurotrophic factor expression. *J Int Med Res* 2019; 47:2471–2482. 31014147 10.1177/0300060519843738 PMC6567697

[13] Rozet I Metzner J Brown M. Dexmedetomidine does not affect evoked potentials during spine surgery. *Anesth Analg* 2015; 121:492–501. 26097987 10.1213/ANE.0000000000000840

[14] Tsujikawa S Ikeshita K. Low-dose dexmedetomidine provides hemodynamics stabilization during emergence and recovery from general anesthesia in patients undergoing carotid endarterectomy: a randomized double-blind, placebo-controlled trial. *J Anesth* 2019; 33:266–272. 30656404 10.1007/s00540-019-02612-w

[15] Le Guen M Liu N Tounou F. Dexmedetomidine reduces propofol and remifentanil requirements during bispectral index-guided closed-loop anesthesia: a double-blind, placebo-controlled trial. *Anesth Analg* 2014; 118:946–955. 24722260 10.1213/ANE.0000000000000185

[16] Liu Y Liang F Liu X. Dexmedetomidine reduces perioperative opioid consumption and postoperative pain intensity in neurosurgery: a meta-analysis. *J Neurosurg Anesthesiol* 2018; 30:146–155. 28079737 10.1097/ANA.0000000000000403

[17] Jang YE Kim YC Yoon HK. A randomized controlled trial of the effect of preoperative dexmedetomidine on the half maximal effective concentration of propofol for successful i-gel insertion without muscle relaxants. *J Anesth* 2015; 29:338–345. 25394762 10.1007/s00540-014-1949-9

[18] Peden CJ Cloote AH Stratford N Prys-Roberts C. The effect of intravenous dexmedetomidine premedication on the dose requirement of propofol to induce loss of consciousness in patients receiving alfentanil. *Anaesthesia* 2001; 56:408–413. 11350323 10.1046/j.1365-2044.2001.01553.x

[19] Pereira JV Sanjanwala RM Mohammed MK. Dexmedetomidine versus propofol sedation in reducing delirium among older adults in the ICU: a systematic review and meta-analysis. *Eur J Anaesthesiol* 2020; 37:121–131. 31860605 10.1097/EJA.0000000000001131

[20] Su X Meng ZT Wu XH. Dexmedetomidine for prevention of delirium in elderly patients after noncardiac surgery: a randomised, double-blind, placebo-controlled trial. *Lancet* 2016; 388:1893–1902. 27542303 10.1016/S0140-6736(16)30580-3

[21] Kim E Kim HC Lee S. Dexmedetomidine confers neuroprotection against transient global cerebral ischemia/reperfusion injury in rats by inhibiting inflammation through inactivation of the TLR-4/NF-κB pathway. *Neurosci Lett* 2017; 649:20–27. 28392361 10.1016/j.neulet.2017.04.011

[22] Luo C Ouyang MW Fang YY. Dexmedetomidine protects mouse brain from ischemia-reperfusion injury via inhibiting neuronal autophagy through up-regulating HIF-1α. *Front Cell Neurosci* 2017; 11:197. 28729825 10.3389/fncel.2017.00197 PMC5498477

[23] Zeng X Wang H Xing X. Dexmedetomidine protects against transient global cerebral ischemia/reperfusion induced oxidative stress and inflammation in diabetic rats. *PLoS One* 2016; 11:e0151620. 26982373 10.1371/journal.pone.0151620 PMC4794239

[24] Bloor BC Ward DS Belleville JP Maze M. Effects of intravenous dexmedetomidine in humans. II. Hemodynamic changes. *Anesthesiology* 1992; 77:1134–1142. 1361311 10.1097/00000542-199212000-00014

[25] Ebert TJ Hall JE Barney JA. The effects of increasing plasma concentrations of dexmedetomidine in humans. *Anesthesiology* 2000; 93:382–394. 10910487 10.1097/00000542-200008000-00016

[26] Huupponen E Maksimow A Lapinlampi P. Electroencephalogram spindle activity during dexmedetomidine sedation and physiological sleep. *Acta Anaesthesiol Scand* 2008; 52:289–294. 18005372 10.1111/j.1399-6576.2007.01537.x

[27] Seidel K Jeschko J Schucht P. Somatosensory evoked potential and transcranial Doppler monitoring to guide shunting in carotid endarterectomy. *J Neurol Surg A Cent Eur Neurosurg* 2021; 82:299–307. 31935785 10.1055/s-0039-1698441

[28] Schnider TW Minto CF Shafer SL. The influence of age on propofol pharmacodynamics. *Anesthesiology* 1999; 90:1502–1516. 10360845 10.1097/00000542-199906000-00003

[29] Jain AR Bellolio MF Stead LG. Treatment of hypertension in acute ischemic stroke. *Curr Treat Options Neurol* 2009; 11:120–125. 19210914 10.1007/s11940-009-0015-7

[30] Ely EW Margolin R Francis J. Evaluation of delirium in critically ill patients: validation of the confusion assessment method for the intensive care unit (CAM-ICU). *Crit Care Med* 2001; 29:1370–1379. 11445689 10.1097/00003246-200107000-00012

[31] R Foundation for Statistical Computing, Team RC. A language and environment for statistical computing. 2020.

[32] Xiong M Zheng Z Hu ZR. Propofol-sparing effect of different concentrations of dexmedetomidine: Comparison of gender differences. *Anaesthesist* 2019; 68:15–21. 10.1007/s00101-018-0506-6 PMC6342900 30406275

[33] Kang WS Kim SY Son JC. The effect of dexmedetomidine on the adjuvant propofol requirement and intraoperative hemodynamics during remifentanil-based anesthesia. *Korean J Anesthesiol* 2012; 62:113–118. 22379564 10.4097/kjae.2012.62.2.113 PMC3284731

[34] Purdon PL Sampson A Pavone KJ Brown EN. Clinical electroencephalography for anesthesiologists: part I: background and basic signatures. *Anesthesiology* 2015; 123:937–960. 26275092 10.1097/ALN.0000000000000841 PMC4573341

[35] Talke P Lobo E Brown R. Systemically administered alpha2-agonist-induced peripheral vasoconstriction in humans. *Anesthesiology* 2003; 99:65–70. 12826844 10.1097/00000542-200307000-00014

[36] Cioccari L Luethi N Bailey M. The effect of dexmedetomidine on vasopressor requirements in patients with septic shock: a subgroup analysis of the Sedation Practice in Intensive Care Evaluation [SPICE III] Trial. *Crit Care* 2020; 24:441. 32678054 10.1186/s13054-020-03115-x PMC7367420

[37] Ascher E Markevich N Schutzer RW. Cerebral hyperperfusion syndrome after carotid endarterectomy: predictive factors and hemodynamic changes. *J Vasc Surg* 2003; 37:769–777. 12663976 10.1067/mva.2003.231

[38] Hu J Zhu M Gao Z. Dexmedetomidine for prevention of postoperative delirium in older adults undergoing oesophagectomy with total intravenous anaesthesia: a double-blind, randomised clinical trial. *Eur J Anaesthesiol* 2021; 38:S9–s17. 33122571 10.1097/EJA.0000000000001382

[39] Liaquat Z Xu X Zilundu PLM. The current role of dexmedetomidine as neuroprotective agent: an updated review. *Brain Sci* 2021; 11:846. 34202110 10.3390/brainsci11070846 PMC8301952

[40] Walker CT Kim HJ Park P. Neuroanesthesia guidelines for optimizing transcranial motor evoked potential neuromonitoring during deformity and complex spinal surgery: a Delphi consensus Study. *Spine* 2020; 45:911–920. 32539292 10.1097/BRS.0000000000003433

[41] Xiang B Jiao S Zhang Y. Effects of desflurane and sevoflurane on somatosensory-evoked and motor-evoked potential monitoring during neurosurgery: a randomized controlled trial. *BMC Anesthesiol* 2021; 21:240. 34620093 10.1186/s12871-021-01463-x PMC8496030

[42] Dulfer SE Groen H Groen RJM. The association of physiological and pharmacological anesthetic parameters with motor-evoked potentials: a multivariable longitudinal mixed model analysis. *Anesth Analg* 2023; 139:609–616. 38153871 10.1213/ANE.0000000000006757 PMC11305622

[43] Nathan N Tabaraud F Lacroix F. Influence of propofol concentrations on multipulse transcranial motor evoked potentials. *Br J Anaesth* 2003; 91:493–497. 14504148 10.1093/bja/aeg211

[44] Mahmoud M Sadhasivam S Salisbury S. Susceptibility of transcranial electric motor-evoked potentials to varying targeted blood levels of dexmedetomidine during spine surgery. *Anesthesiology* 2010; 112:1364–1373. 20460997 10.1097/ALN.0b013e3181d74f55

[45] Andleeb R Agrawal S Gupta P. Evaluation of the effect of continuous infusion of dexmedetomidine or a subanesthetic dose ketamine on transcranial electrical motor evoked potentials in adult patients undergoing elective spine surgery under total intravenous anesthesia: a randomized controlled exploratory study. *Asian Spine J* 2022; 16:221–230. 34407570 10.31616/asj.2021.0015 PMC9066250

